# Activated Lymphocyte-Derived DNA Drives Glucose Metabolic Adaptation for Inducing Macrophage Inflammatory Response in Systemic Lupus Erythematosus

**DOI:** 10.3390/cells12162093

**Published:** 2023-08-18

**Authors:** Hanqing Zhao, Zhenke Wen, Sidong Xiong

**Affiliations:** Jiangsu Key Laboratory of Infection and Immunity, Institutes of Biology and Medical Sciences, Soochow University, Suzhou 215123, China

**Keywords:** ALD-DNA, SLE, macrophage inflammatory response, glucose metabolism, cAMP

## Abstract

Activated lymphocyte-derived DNA (ALD-DNA) has been reported to drive the polarization of macrophages toward M2b, producing inflammatory cytokines and inducing inflammation, correspondingly playing an essential role in the development of systemic lupus erythematosus (SLE). Recently, accumulating evidence has pinpointed metabolic adaptation as the crucial cell-intrinsic determinant for inflammatory response, in which glucose metabolism is the key event. However, whether and how glucose metabolism was involved in ALD-DNA-induced macrophage inflammatory response and SLE development remains unclear. Herein, we performed glucose metabolomic analyses of ALD-DNA-stimulated macrophages and uncovered increased glycolysis and diminished pentose phosphate pathway (PPP), as well as enhanced glycogenesis. In ALD-DNA-stimulated macrophages, increased glycolysis resulted in higher lactate production, whereas diminished PPP efficiently led to lower levels of nicotinamide adenine dinucleotide phosphate (NADPH) with higher levels of reactive oxygen species (ROS). While blockade of lactate generation exerted no significant effect on macrophage inflammation in response to ALD-DNA, scavenging ROS fundamentally inhibited the inflammatory response of ALD-DNA-stimulated macrophages. Further, cyclic adenosine monophosphate (cAMP), a master for regulating glycogen metabolism, was downregulated by ALD-DNA in macrophages, which subsequently imbalanced glycogen metabolism toward glycogenesis but not glycogenolysis. Administration of cAMP effectively restored glycogenolysis and enhanced PPP, which correspondingly reduced ROS levels and inhibited the inflammatory response of ALD-DNA-stimulated macrophages. Finally, blocking glucose metabolism using 2-deoxy-D-glucose (2-DG) efficiently restricted macrophage inflammatory response and alleviated ALD-DNA-induced lupus disease. Together, our findings demonstrate that ALD-DNA drives the adaptation of glucose metabolism for inducing macrophage inflammatory response in SLE, which might further our understanding of disease pathogenesis and provide clues for interventive explorations.

## 1. Introduction

Systemic lupus erythematosus (SLE) is an autoimmune disease with complex and diverse clinical manifestations. The accumulation of immunogenic Self-DNA and IgG anti-dsDNA antibodies is a common feature of SLE patients, which is closely related to the pathogenesis of the disease and is an important biomarker for SLE diagnosis [1]. Normally, DNA is strictly localized to the nucleus and mitochondria, but due to the excessive apoptotic response in SLE patients, highly immunogenic Self-DNA is released from the cells [2]. Such Self-DNA activates innate immune cells via DNA sensors and produces type I interferons as well as various pro-inflammatory cytokines, driving the onset and relapse of SLE [3].

Our previous studies have demonstrated that activated lymphocyte-derived DNA (ALD-DNA) can effectively induce macrophage M2b polarization, exacerbating the tissue inflammatory response [4,5,6,7,8]. Specifically, extracellular high mobility group box-1 protein (HMGB1) facilitates the lysosomal accumulation of ALD-DNA, which subsequently activates toll-like receptor 9 (TLR9) signaling for inducing inflammatory response [7,9]. ALD-DNA can be sensed by the DNA-dependent activator of interferon-regulatory factors (DAI), inducing inflammatory cytokines from the M2b macrophages [8]. Furthermore, ALD-DNA can induce Notch1 signaling for instructing macrophage inflammation, and serum amyloid P component (SAP) modulates ALD-DNA-induced inflammation through regulating phosphoinositide 3-kinase (PI3K)-protein kinase B (Akt)-extracellular signal-regulated kinase (ERK) pathway [5]. Of importance, the removal of macrophages reduces the severity of ALD-DNA-induced lupus [10], assigning an essential role to ALD-DNA-induced macrophage inflammation in SLE pathogenesis.

Macrophages are highly heterogeneous immune cells, and metabolic adaptation can profoundly affect the phenotype and function of macrophages during the immune response [11]. Notch signaling is well-defined in activating the PI3K/AKT pathway and subsequent glucose uptake [12], and TLR9 signaling promotes glucose uptake through the regulation of mTORC1 activity [13]. Of interest, recent evidence pinpoints a synergistic function of TLR9 and Notch1 in driving the metabolic adaptation for populating cancer stem-like cells [14], indicating that ALD-DNA-induced TLR9 and Notch1 signaling might drive metabolic adaptation in macrophages. Of importance, glucose metabolism is a key metabolic event in macrophage inflammation [11]. The glycolytic enzyme pyruvate kinase M2 (PKM2) promotes activation of the inflammasome and enhances pro-IL-1β expression through activation of eukaryotic translation initiation factor 2 alpha kinase 2 (EIF2AK2) [15,16]. Fructose-2,6-bisphosphatase 3 (PFKFB3) promotes the uptake and elimination of virus-infected cells by metabolically supporting the antiviral capacity of macrophages [17]. Glycogen metabolism in macrophages activates the UDPG-P2Y14 signaling pathway to initiate an inflammatory response [18]. In addition, macrophages have been reported to drive their inflammatory phenotype via the pentose phosphate pathway (PPP) and mitochondrial succinate oxidation [19,20]. With regard to macrophage inflammation, there have been many reports on interferon-γ (IFN-γ) and lipopolysaccharide (LPS) induced inflammatory macrophage metabolism, with different macrophage phenotypes and metabolic profiles produced with different inflammatory macrophage stimulators [21,22,23,24]. In SLE, macrophages may exhibit different cellular metabolic profiles during the uptake and clearance of autoantigens, profoundly affecting macrophage phenotype and function. However, whether and how glucose metabolism might be involved in ALD-DNA-induced macrophage inflammation remain undefined.

In this study, we characterized the cellular glucose metabolic profile of macrophages after ALD-DNA stimulation, aiming to explore whether and how glucose metabolic adaptation might be involved in macrophage inflammatory responses in SLE. Together, we provide evidence that glucose metabolism is involved in controlling the inflammatory response of macrophages in response to ALD-DNA, serving as a therapeutic target for the treatment of SLE.

## 2. Materials and Methods

### 2.1. Mice

Six-to-eight-week-old female BALB/c mice were purchased from the Experimental Animal Center of the Chinese Academy of Sciences (Shanghai, China). Mice were housed in a specific pathogen-free room under controlled temperature and humidity. All mice procedures were conducted according to the Institutional Guide for the Care and Use of Medical Laboratory Animals and approved by the Ethics Committee of Soochow University (202107A0442).

### 2.2. DNA Preparation

ALD-DNA was prepared with murine splenocytes from six-week-old female BALB/c mice and cultured with Con A (Sigma-Aldrich, St. Louis, MO, USA) in vitro as previously described [4,10,25]. For generation of ALD-DNA, splenocytes were seeded at 2 × 10^6^ cells/mL in 75 cm^2^ cell culture flask and cultured in the presence of Con A (5 µg/mL) for 6 days to induce apoptosis. Briefly, genomic DNAs were purified using the DNeasy Blood and Tissue Kits (Qiagen, Hilden, Germany) after the treatment of S1 nuclease (Takara, San Jose, CA, USA) and proteinase K (Sigma-Aldrich, St. Louis, MO, USA). To exclude LPS contamination, sterile endotoxin-free plastic ware and reagents were used for DNA preparation. The OD260/280 and concentration of DNA were detected by a microplate reader (Bio-TEK), and the OD260/280 of DNA used in the experiment was between 1.8 and 2.0.

### 2.3. Cell Culture

RAW264.7 cells from the American Type Culture Collection (ATCC, Manassas, VA, USA) were cultured in DMEM (Hyclone, Logan, UT, USA) supplemented with 10% FBS (Eallbio, Beijing, China) and 100 U/mL penicillin/streptomycin (Invitrogen, Waltham, MA, USA) in 5% CO_2_ incubator at 37 °C. Such macrophages were stimulated with ALD-DNA (50 μg/mL) and detected the metabolic adaptation and inflammatory response.

### 2.4. Metabolite Profiling

Intracellular metabolites were extracted, and metabolite profiles were obtained using LC-MS/MS coupling technique. Separation was performed by high-performance liquid chromatography (Shimadzu, Kyoto, Japan), and mass spectrometry was performed using the QTRAP5500 mass spectrometer (AB SCIEX, Framingham, MA, USA) in positive/negative ion mode. Multiple reaction monitoring (MRM) mode was used to detect the ion pairs to be tested. The peak area and retention time were extracted by Multi-Quant V3.0.3 software. The retention time was corrected by metabolite standard, and metabolite identification was performed. The peak area of metabolite-extracted ions was normalized by internal standard L-Glutamate_D5. Metabolite levels were normalized to protein content, and all data have been deposited in the OMIX (ID OMIX004242), China National Center for Bioinformation.

### 2.5. Glucose Uptake Assay

Cells were incubated with ALD-DNA for 0, 6, 12, and 24 h, followed by replacing the medium with a sugar-free medium containing 10% fetal bovine serum and cultured in a carbon dioxide incubator for 30 min. Then, 2-NBDG (APExBIO, Houston, TX, USA) with a final concentration of 100 μM was added and cultured in a carbon dioxide incubator for 15 min. After that, cells were collected and washed twice with PBS and then detected by flow cytometry (BD Biosciences, Dubai, United Arab Emirates) within 10 min.

### 2.6. Measurement of Lactate Production

Cells were seeded in a 6-well plate and incubated with ALD-DNA for 0, 6, 12, and 24 h. Lactate concentrations in the culture media were measured using the Lactate Assay Kit (JianCheng, Nanjing, China) according to the manufacturer’s instructions. For the detection of lactate, 450 nm was used using a microplate reader (Bio-TEK, Winooski, VT, USA). Lactate content was normalized to cell numbers.

### 2.7. Real-Time PCR Analysis

Total RNA was extracted from cells with TRIzol reagent (Invitrogen Life Technologies, Carlsbad, CA, USA) according to the manufacturer’s instructions. The cDNA was synthesized with the PrimeScript RT reagent kit (Takara Bio). The expression of the gene encoding interleukin-1 beta (*Il1β*), interleukin-6 (*Il6*), interleukin-10 (*Il10*), glucose transporter type 1 (*Glut1*), glucose-6-phosphate dehydrogenase (*G6pd*), hypoxia-inducible factor 1 subunit alpha (*Hif1a*), lactate dehydrogenase A (*Ldha*), hexokinase 2 (*Hk2*), and phosphoglucomutase 1 (*Pgm1*) was quantified by real-time PCR using SYBR Green system (ABI), following the manufacturer’s protocol. All gene expressions were normalized to that of the housekeeping gene actin beta (*Actb*), and the primers were as follows:

*Il1b* F-5′-GCAACTGTTCCTGAACTCAACT-3′, R-5′-ATCTTTTGGGGTCCGTCAACT;

*Il6* F-5′-TAGTCCTTCCTACCCCAATTTCC-3′, R-5′-TTGGTCCTTAGCCACTCCTTC-3′;

*Il10* F-5′-GCTCTTACTGACTGGCATGAG-3′, R-5′-CGCAGCTCTAGGAGCATGTG-3′;

*Ldha* F-5′-TGTCTCCAGCAAAGACTACTGT3′, R-5′-GACTGTACTTGACAATGTTGGGA-3′;

*Glut1* F-5′-GCAGTTCGGCTATAACACTGG-3′, R-5′-GCGGTGGTTCCATGTTTGATTG-3′;

*Hk2* F-5′-ATCGCCGGATTGGAACAGAA-3′, R-5′-ATGTCAAAGTCCCCTCTGCG-3′;

*Pgm1* F-5′-AGCCAATGACCCAGATGCTGAC-3′, R-5′TCCAGGAAGTGAAGAGCCACCA-3′;

*G6pd* F-5′-GACCAAGAAGCCTGGCATGTTC-3′, R-5′-AGACATCCAGGATGAGGCGTTC-3′;

*Actb* F-5′-GGCTGTATTCCCCTCCATCG-3′, R-5′-CCAGTTGGTAACAATGCCATGT-3′.

### 2.8. Enzyme-Linked Immunosorbent Assay (ELISA) Assay

To assess protein levels of IL-1β and IL-6 in the cell culture supernatants, ELISA assays were performed with IL-1β Mouse ELISA Kit and IL-6 Mouse ELISA Kit (eBioscience, San Diego, CA, USA) according to the manufacturer’s instructions.

### 2.9. Western Blot Analysis

Cells were lysed in cell lysis buffer containing a protease inhibitor cocktail at 4 °C for 30 min. The lysates were denatured, subjected to SDS-PAGE, and transferred to PVDF membranes (Thermo, Waltham, MA, USA). The membranes were blocked and reacted with primary antibodies overnight at 4 °C. Horseradish peroxidase (HRP)-conjugated secondary antibodies (AS003, ABclonal, Woburn, MA, USA) were used for subsequent incubation for 2 h at room temperature. The protein bands were visualized using the enhanced chemiluminescence (ECL) system (Amersham Imager 600). Specifically, such immunoblots were performed with the following antibodies: anti-Glycogen Synthase 1 (GYS1) Rabbit pAb (A2519, ABclonal, Woburn, MA, USA); anti-Glycogen Phosphorylase (PYGL) Rabbit pAb (A6710, ABclonal, Woburn, MA, USA); and anti-β-tubulin mAb (M20005, Abmart, Shanghai, China)

### 2.10. GSH:GSSG Ratio

Reduced glutathione (GSH) and oxidized glutathione (GSSG) levels were determined using a GSSG and GSH Assay Kit (Beyotime, Shanghai, China) according to the manufacturer’s instructions. For the detection of glutathione, 412 nm was used with a microplate reader (Bio-TEK). The GSH:GSSG ratio was calculated using the following equation: GSH/GSSG = [Total GSH − (2 × GSSG)]/GSSG.

### 2.11. ROS Assay

ROS levels were determined using Reactive Oxygen Species Assay Kit (Beyotime, Shanghai, China) according to the manufacturer’s instructions. Cells were loaded with 10 nM DCFH-DA for 20 min at 37 °C, protected from light. Cells were then washed, scraped in PBS, and immediately analyzed by flow cytometry (BD Biosciences, Dubai, United Arab Emirates) using 488 nm excitation for the DCFH.

### 2.12. Glycogen Assay

Glycogen was determined using the Glycogen Assay Kit (JianCheng, Nanjing, China) and Glycogen Periodic Acid Schiff (PAS) Stain Kit (Solarbio, Beijing, China), respectively, according to the manufacturer’s Instructions.

### 2.13. SLE Model and 2-DG Treatment

For the induction of the SLE model, six- to eight-week-old female BALB/c mice were randomly divided into several groups and immunized by subcutaneous injection with 0.2 mL of ALD-DNA (50 μg/mouse) plus complete Freund’s adjuvant (CFA, Sigma, St. Louis, MO, USA) on week 0, followed by injections with 0.2 mL of ALD-DNA (50 μg/mouse) plus incomplete Freund’s adjuvant (IFA, Sigma, St. Louis, MO, USA) on week 2 and week 4. Control mice received an equal volume of CFA plus PBS. After the initial immunization, the glucose analog 2-DG treatment was performed for 8 weeks with 2-DG (5 mg/mL) dissolved in drinking water. Eight weeks after the initial immunization, mice were sacrificed for detection of the disease development.

### 2.14. Flow Cytometry Analysis and Cell Sorting

Murine renal tissues were surgically resected and dispersed in RPMI 1640 containing 10% FBS and 1 mg/mL collagenase Ⅳ (Sigma-Aldrich, St. Louis, MO, USA) at 37 °C for 1 h, followed by progressive sieving to obtain single-cell suspensions. FITC-labeled anti-F4/80 (BD Biosciences, Dubai, United Arab Emirates) were incubated with renal cells at 4 °C for 30 min, and F4/80^+^ macrophages were analyzed and sorted by flow cytometry (BD Biosciences, Dubai, United Arab Emirates).

### 2.15. Histology

Hematoxylin-eosin (H&E) staining of renal tissue was performed on 5 μm paraffin sections. The kidney score of glomerulonephritis was determined by using the ISN/RPS2003 classification [26]. Immune-fluorescent staining of renal tissue cryosections was performed using FITC-conjugated goat anti-mouse IgG Ab (Sigma-Aldrich, St. Louis, MO, USA).

### 2.16. Anti-dsDNA Antibodies and Urinary Protein Assay

Serum samples were determined by IgG anti-dsDNA ELISA kit (Alpha Diagnostic, San Antonio, TX, USA) for the presence of anti-dsDNA antibodies according to the manufacturer’s instructions. Urinary protein was measured with the Bicinchoninic acid assay (BCA) Protein Assay Kit (ThermoFisher Scientific, Waltham, MA, USA) according to the manufacturer’s instructions.

### 2.17. Statistical Analysis

All data are expressed as means ± SEM. The statistical significance of the differences in the experimental data was valued by the Student’s *t*-test and one-way ANOVA. All the analyses were conducted using the GraphPad Prism V 9.0 software. Statistical significance levels were set as * *p* < 0.05, ** *p* < 0.01, *** *p* < 0.001, and **** *p* < 0.0001.

## 3. Results

### 3.1. ALD-DNA Induces an Inflammatory Response with Glucose Metabolic Adaptation

**Figure 1:**
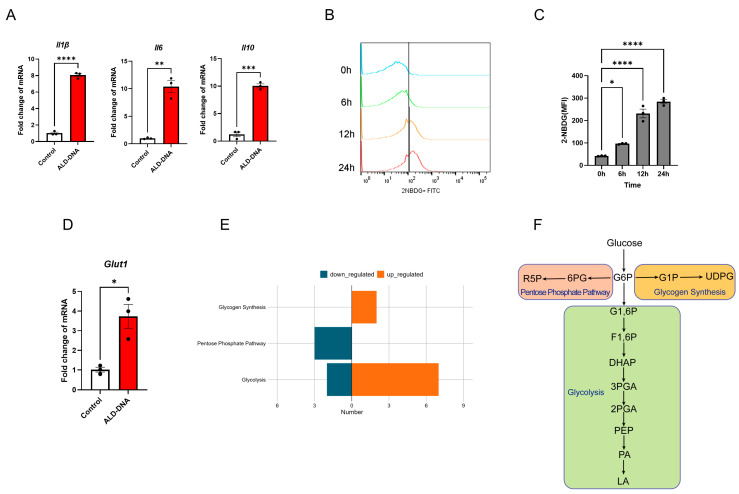
ALD-DNA induces adaptation of glucose metabolism in macrophages. (**A**) RAW264.7 cells were stimulated with ALD-DNA (50 μg/mL) or control (PBS) for 12 h and detected for mRNA levels of *Il1β*, *Il6*, and *Il10* using qPCR. (**B**,**C**) RAW264.7 cells were stimulated with ALD-DNA (50 μg/mL) for the indicated time. RAW264.7 cells were co-incubated with 2NBDG (100 nM) for 15 min at the indicated time, and fluorescence intensity was detected by flow cytometry. Representative and collective mean fluorescence intensity (MFI) from 3 independent experiments. (**D**) RAW264.7 cells were stimulated with ALD-DNA (50 μg/mL) or control (PBS) for 12 h. (**E**) LC-MS/MS showing the intracellular metabolites in RAW264.7 cells after stimulation with or without ALD-DNA (50 μg/mL) for 12 h. (**F**) Schematic of glucose metabolism ribulose 5-phosphate (R5P), 6-phosphogluconic acid (6PG), glucose-1-phosphate (G6P), glucose-1-phosphate (G1P), UDP-glucose (UDPG), glucose 1,6-biphosphate (G1,6P), fructose-1,6-bisphosphate (F1,6P), dihydroxyacetone phosphate (DHAP), 3-phosphoglycerate (3PGA), 2-phosphoglycerate (2PGA), phosphoenolpyruvic acid (PEP), pyruvic acid (PA), lactic acid (LA). Data were mean ± SEM of 3 independent experiments. * *p* < 0.05, ** *p* < 0.01, *** *p* < 0.001, **** *p* < 0.0001 with paired *t*-test (**A**,**D**) and ANOVA plus Turkey method (**C**).

While ALD-DNA-induced macrophage inflammation is crucial for SLE development and glucose metabolism has been implicated in macrophage function [27], whether and how ALD-DNA might shape the glucose metabolism in inflammatory response remains unclear. Herein, we observed that ALD-DNA efficiently induced the generation of inflammatory cytokines from macrophages (Figure 1A). Of interest, stimulation with ALD-DNA promoted the glucose uptake of macrophages (Figure 1B,C). Further, we found the up-regulated transcription of *Glut1* of ALD-DNA-stimulated macrophages (Figure 1D).

To characterize the glucose metabolic features of ALD-DNA-stimulated macrophages, we performed a metabolomic analysis for detecting the glucose metabolites. While the glucose flux into three metabolic pathways involved glycolysis and pentose phosphate pathway (PPP) (Figure 1E), we identified enhanced glycogen synthesis and glycolysis, together with diminished PPP, in macrophages in response to ALD-DNA (Figure 1F). Together, these results suggest that ALD-DNA efficiently drives glucose metabolic adaptation in macrophages.

### 3.2. Glycolysis Exerts no Significant Effect on ALD-DNA-Induced Inflammation

**Figure 2:**
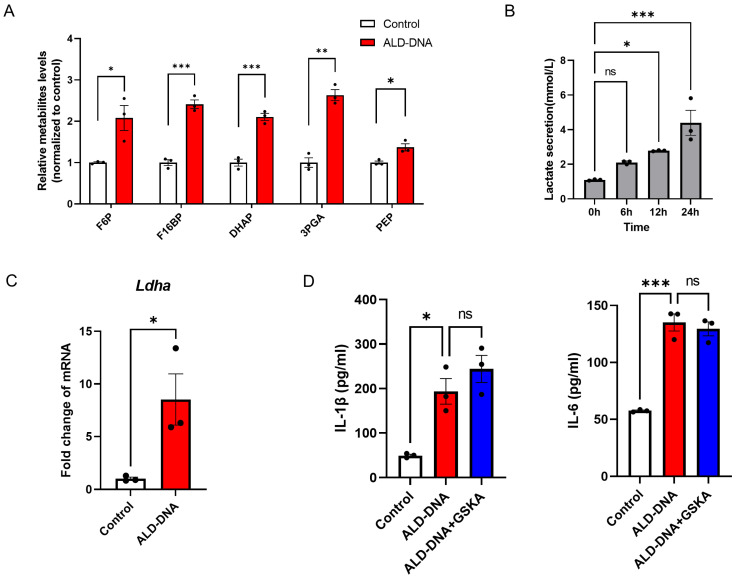
Lactate is not required for ALD-DNA-induced inflammatory response. (**A**) RAW264.7 cells were stimulated with ALD-DNA or control (PBS) for 12 h. Levels of glycolysis intermediates fructose-6-phosphate (F6P), fructose 1,6-bisphosphate (F16BP), dihydroxyacetone phosphate (DHAP), 3-phosphoglycerate (3PGA), and phosphoenolpyruvic acid (PEP) were determined by LC-MS/MS. (**B**) RAW264.7 cells were stimulated with ALD-DNA for the indicated time and determined the lactate levels in the culture medium. (**C**) RAW264.7 cells were stimulated with ALD-DNA or control (PBS) for 12 h. (**D**) RAW264.7 cells were stimulated with ALD-DNA ± GSKA (10 μM) for 24 h, and measured for IL-1β plus IL-6 in the culture supernatants. Data are mean ± SEM of 3 independent experiments. ns, no significance. * *p* < 0.05, ** *p* < 0.01, *** *p* < 0.001 with paired *t*-test (**A**,**C**) and ANOVA plus Turkey method (**B**,**D**).

The glycolytic intermediate metabolites were significantly increased in macrophages in response to ALD-DNA (Figure 2A), suggesting an increased level of glycolysis. In line with such findings, ALD-DNA stimulation promoted the generation of lactate from macrophages (Figure 2B). To understand how ALD-DNA enhanced glycolysis, we detected the key enzyme LDHA and observed higher expression levels in ALD-DNA-stimulated macrophages (Figure 2C).

To evaluate the possible role of glycolysis in ALD-DNA-induced inflammation, macrophages were incubated with ALD-DNA plus LDHA inhibitor GSKA. We found that GSKA exerted no significant effect on the production of IL-1β and IL-6 from ALD-DNA-stimulated macrophages (Figure 2D), indicating that glycolytic lactate is not essential for ALD-DNA-induced inflammation.

### 3.3. PPP^low^ Results in ROS^high^ for Licensing ALD-DNA-Induced Inflammatory Response

**Figure 3:**
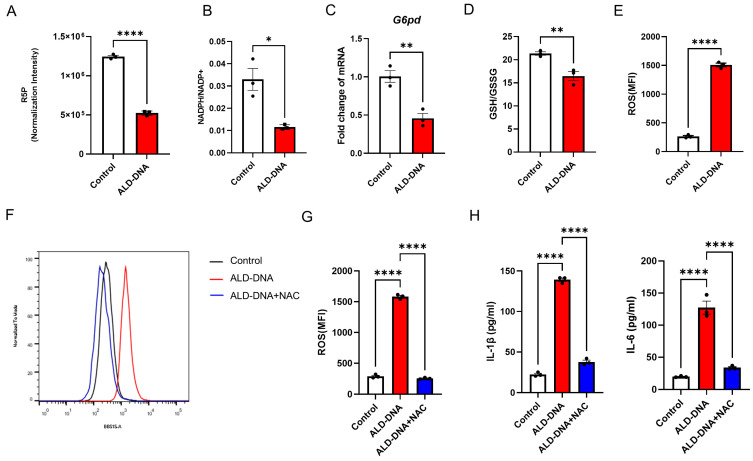
PPP^low^ and ROS^high^ in ALD-DNA-induced inflammatory response. RAW264.7 cells were stimulated with ALD-DNA or control (PBS) for 12 h. R5P (**A**) and NADPH/NADP+ ratio (**B**) were determined by LC-MS/MS. mRNA levels of *G6pd* (**C**) were determined by qPCR. Intracellular GSH:GSSG ratio (**D**) was determined by the GSSG and GSH assay kit. Intracellular ROS (**E**) was determined by flow cytometry. ROS levels in response to treatment with NAC (**F**,**G**, 5 mM). Levels of IL-1β and IL-6 in the culture supernatants (**H**) were measured by ELISA. Data are mean ± SEM of 3 independent experiments. * *p* < 0.05, ** *p* < 0.01, **** *p* < 0.0001 with paired *t*-test (**A**–**E**) and ANOVA plus Turkey method (**G**,**H**).

We found decreased levels of R5P and NADPH in ALD-DNA-stimulated macrophages (Figure 3A,B), suggesting an impaired PPP in those macrophages. In support, the transcription of *G6pd* is significantly inhibited in ALD-DNA-stimulated macrophages (Figure 3C).

NADPH is well-known as an essential cofactor of glutathione reductase, playing a key role in cellular antioxidation systems. Consistent with the decreased generation of NADPH, lower levels of the GSH:GSSG ratio and higher levels of ROS were observed in ALD-DNA-stimulated macrophages (Figure 3D,E). Of importance, N-acetylcysteine (NAC), a ROS scavenger, fundamentally reduced the ROS levels in ALD-DNA-stimulated macrophages (Figure 3F,G), leading to diminished inflammatory response (Figure 3H). These results suggest that the PPP-related ROS might be critically involved in the ALD-DNA-induced inflammation of macrophages.

### 3.4. ALD-DNA Drives ROS^high^ by Reducing cAMP in Macrophages

**Figure 4:**
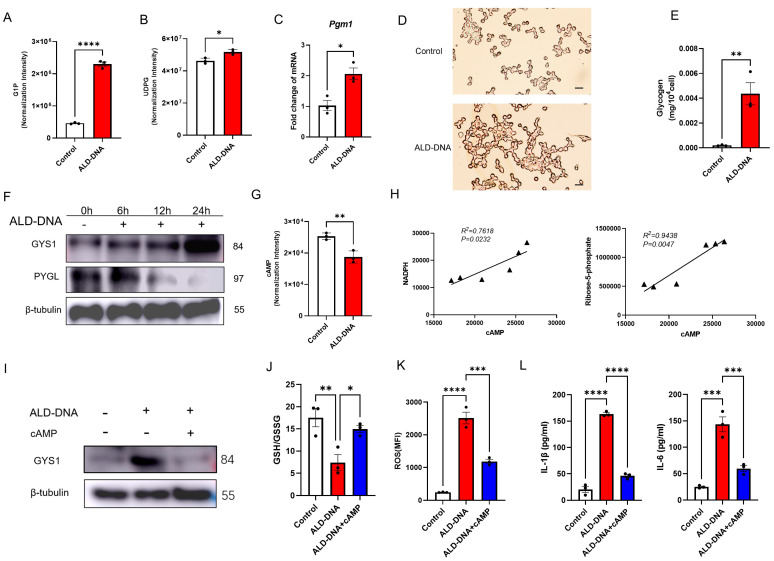
Decreased cAMP and unbalanced glycogen metabolism in ALD-DNA-induced inflammatory response. (**A**–**E**) RAW264.7 cells were stimulated with ALD-DNA or control (PBS) for 12 h. (**A**) G1P and (**B**) UDPG were determined via LC-MS/MS. (**C**) mRNA levels of *Pgm1* in the RAW264.7 cells were determined by qPCR. (**D**) PAS staining was performed for intracellular glycogen in macrophages. Scar bar, 100 μm. (**E**) The intracellular glycogen of macrophages was examined with a glycogen detection kit. (**F**) RAW264.7 were stimulated with ALD-DNA for the indicated time. GYS1 and PYGL protein levels were determined by immunoblots. (**G**) RAW264.7 cells were stimulated with ALD-DNA or control (PBS) for 12 h. (**H**) Correlations of PPP-associated metabolites and cAMP in RAW264.7 cells in response to ALD-DNA. (**I**–**K**) RAW264.7 cells were stimulated with ALD-DNA ± 8-Br-cAMP (50 μM) for 12 h. (**L**) RAW264.7 cells were stimulated with ALD-DNA ± 8-Br-cAMP (50μM) for 24 h. Data are mean ± SEM of 3 independent experiments. * *p* < 0.05, ** *p* < 0.01, *** *p* < 0.001, **** *p* < 0.0001 with paired *t*-test (**A**–**G**), Pearson analysis (**H**), and ANOVA plus Turkey method (**J**–**L**).

It has been reported that IFN-γ/LPS treatment stimulates macrophages to synthesize glycogen, followed by glycogenolysis that generates G6P and fuels PPP for inflammatory macrophage survival [18]. Herein, we found increased levels of G1P and UDPG, which were accompanied by the upregulated transcription of *Pgm1* in macrophages in response to ALD-DNA (Figure 4A–C), suggesting a robust glycogen synthesis. In consistency, we examined the glycogen content in ALD-DNA-stimulated macrophages and found a large amount of intracellular glycogen accumulation (Figure 4D,E). Further, we found that ALD-DNA could promote the protein levels of GYS1 while reducing the PYGL in macrophages (Figure 4F). These findings strongly demonstrate an enhanced glycogen synthesis in ALD-DNA-stimulated macrophages.

cAMP is crucially involved in glycogen metabolism, leading to the activation of protein kinase A that, in turn, promotes glycogenolysis and inhibits glycogen synthesis [28,29]. We found that the level of cAMP was decreased in ALD-DNA-stimulated macrophages (Figure 4G), in line with the glycogen accumulation in those macrophages. Such impaired glycogenesis might result in insufficient G6P as the fuel for PPP, driving diminished PPP in ALD-DNA-stimulated macrophages. In support, intracellular cAMP levels were positively correlated with the content of NADPH and R5P in ALD-DNA-stimulated macrophages (Figure 4H).

To determine whether cAMP deficiency was responsible for the robust GYS1 expression and diminished PPP in ALD-DNA-stimulated macrophages, RAW264.7 cells were stimulated with ALD-DNA in the presence or absence of cAMP. We found that cAMP could reduce the protein levels of GYS1 in ALD-DNA-stimulated macrophage (Figure 4I), accompanied by elevated PPP, as evidenced by increased GSH:GSSG ratio and decreased ROS levels (Figure 4J,K). Of importance, we observed that ALD-DNA-induced production of IL-1β and IL-6 was dramatically inhibited by the administration of cAMP (Figure 4L). These results suggest that ALD-DNA stimulation results in decreased cAMP in macrophages, leading to glycogenolysis^low^ and subsequent PPP^low^ for instructing ROS^high^ with the inflammatory response.

### 3.5. Blocking Glucose Metabolism Inhibits ALD-DNA-Induced Inflammatory Response and Lupus Disease

**Figure 5:**
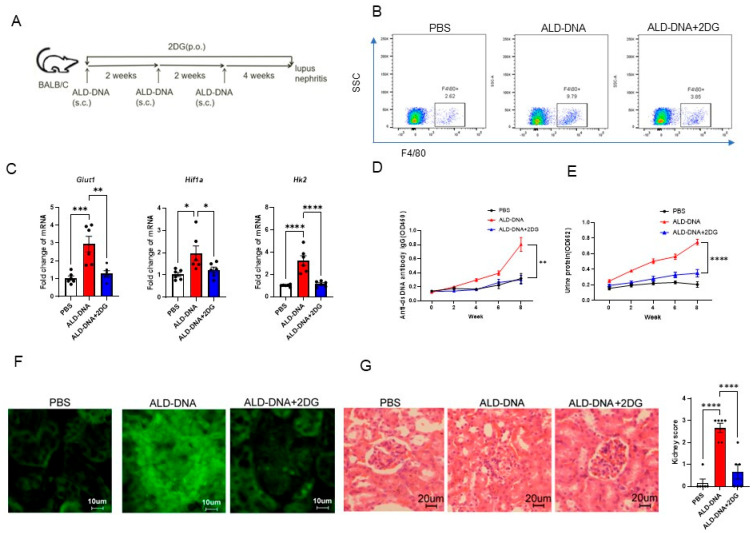
Blocking glucose metabolism abrogates ALD-DNA-induced inflammatory response and lupus disease. (**A**) Female BALB/c mice (*n* = 6 per group) were immunized with ALD-DNA with or without the treatment using glucose analog 2-DG (5 mg/mL dissolved in drinking water for 8 weeks). (**B**) The F4/80^+^ macrophages in the kidney were measured by flow cytometry. (**C**) qPCR analysis of glycolysis-related genes in the renal macrophages. (**D**) The serum IgG anti-dsDNA was measured by ELISA. (**E**) Urine protein levels were assessed by BCA Protein Assay Kit. (**F**) Renal IgG deposition was shown by immunofluorescence staining. (**G**) Nephritic pathological changes were shown by H&E staining. * *p* < 0.05, ** *p* < 0.01, *** *p* < 0.001, **** *p* < 0.0001 with ANOVA plus Turkey method.

Our previous studies have demonstrated that conventional BALB/c mice immunized with ALD-DNA would develop high levels of IgG anti-dsDNA and lupus nephritis, which heavily relies on macrophage inflammatory response [5,6,10]. To test the translational application of targeting glucose metabolism in the treatment of SLE, we established the lupus model by immunizations with ALD-DNA and blocked glucose metabolism with glucose analog 2DG (Figure 5A). In ALD-DNA-induced lupus mice, F4/80^+^ macrophages were increased in the renal tissues, but 2DG treatment reduced such infiltrated macrophages in lupus mice (Figure 5B). Transcript levels of the elevated glucose metabolism genes Hif1a, Hk2, and Glut1 were also repressed by 2DG in those renal macrophages (Figure 5C).

Accordingly, 2-DG significantly inhibited the production of IgG anti-dsDNA, resulting in diminished renal IgG deposition, reduced renal pathological damage, and decreased urinary protein in ALD-DNA-induced lupus mice (Figure 5D–G). In essence, blocking glucose metabolism might be an effective strategy for alleviating lupus disease.

## 4. Discussion

**Figure 6:**
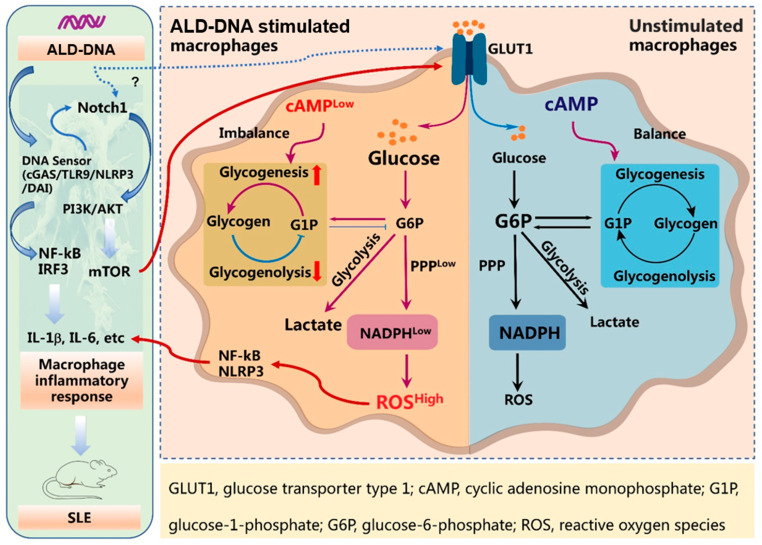
ALD-DNA-stimulated macrophages exert cAMP^low^ and glucose metabolic adaptation for inflammatory response. ALD-DNA triggers DNA sensing through cGAS, TLR9 NLRP3, and DAI, activating Notch1 signaling for inducing macrophage inflammatory responses. Notch1 promotes mTOR activation, which subsequently enhances glucose uptake, instructing the reprogramming of glucose metabolism and thus profoundly affecting macrophage function. In ALD-DNA-stimulated macrophages, cAMP deficiency may lead to robust glycogenesis and impaired glycogenolysis, blocking the G6P flux into the PPP pathway. As a result, ALD-DNA drives NADPH^low^ and subsequent ROS^high^ for inducing inflammatory cytokines.

The metabolic regulation of macrophages has been intensively studied over the past decade, and increasing evidence has identified glucose metabolism as a key player in inflammatory macrophages. In the prototypic autoimmune disease SLE, ALD-DNA drives the polarization of M2b macrophages, inducing inflammatory response and organ damage. In this study, we extended previous studies by demonstrating how ALD-DNA drives glucose metabolic adaptation for inducing inflammatory response (Figure 6). The enhanced glucose uptake of ALD-DNA-stimulated macrophage might be the consequence of activation of TLR9 and Notch1. Of importance, ALD-DNA could downregulate the intracellular level of cAMP, which blocked the glycogenolysis and, thus, impeded the G6P flux into the PPP pathway, resulting in accumulated ROS and subsequent robust inflammatory response. In contrast, although ALD-DNA might enhance the glycolysis of macrophages, promoting the generation of lactate, this was not essential for the production of inflammatory cytokines. Accordingly, blocking glucose metabolism was efficient in alleviating SLE, which might be useful for therapeutic explorations.

Macrophages are a heterogeneous population of immune cells, especially in such autoimmune diseases as SLE, under the influence of multiple factors. Thus, macrophages may exhibit different metabolic network characteristics in response to the effects of different autoantigens, profoundly affecting macrophage function. Cross-linking of IgG immune complexes to the macrophage surface Fcγ receptor leads to increased cellular glycolysis and deepened tissue inflammation, suggesting glycolysis inhibition as a promising strategy to attenuate IgG-associated renal macrophage activation, pro-inflammatory cytokine secretion, and renal inflammation [30]. Self-DNA is widely present in SLE patients, promoting glycolysis and glycogen biosynthesis while decreasing PPP in macrophages. Herein, similar to LPS/IFNγ-driven inflammatory macrophages [31], glycolysis is upregulated in macrophages in response to Self-DNA. Given that Self-DNA is widely accumulating in the circulation and tissues of SLE patients, this might be a crucial resource for inflammatory response through promoting glycogen biosynthesis while inhibiting the PPP in macrophages.

Oxidative stress is activated at various levels in the lupus immune system [32,33]. While reduced glutathione (GSH) is the main intracellular antioxidant defense substance, SLE patients exert reduced GSH levels in erythrocytes, total lymphocytes, and lymphocyte subpopulations [34,35,36]. Accordingly, increasing GSH levels by NAC could reduce the disease severity in patients with SLE [37]. In consequence, mechanisms underlying the decreased GSH are relevant for understanding SLE pathogenies and subsequent therapeutic explorations. Available reports demonstrate that oxidation (8-oxodG, etc.) in SLE patients is the main cause of GSH depletion [38]. Our findings in the current study propose a new insight into reducing GSH in SLE, pinpointing the ALD-DNA-induced imbalance of the cAMP-PPP axis as an important contributor in macrophages.

SLE patients have defects in the adenylate cyclase pathway in PBMCs, including T cells, and lupus-prone MRL/lpr mice also exert low cAMP levels in renal tissues [39,40,41]. In this study, ALD-DNA-stimulated macrophages show decreased cAMP levels, which may block glycogenolysis and lead to glycogen accumulation. Both glycogen synthesis and catabolism are increased in LPS/IFNγ-induced macrophages, and glycogenolysis-derived G6P is a critical glucose flux channeled to the PPP [18]. Consequently, ALD-DNA might downregulate cAMP and impair the glycogenolysis to block the G6P flux for the PPP in macrophages, resulting in diminished NADPH and elevated ROS. Thus, cAMP could be a potential target for the treatment of SLE, both in terms of the reduction in inflammation and the restoration of the antioxidant capacity of macrophages.

In recent decades, extensive studies on immunometabolism have provided us with new insights into the crucial role of intracellular metabolites in immune cells [42,43,44]. Although the metabolic profile of immune cells varies, it is interesting that some metabolic pathways appear to be common to multiple immune cells. In murine SLE, enhanced glycolysis is shared by CD4^+^ T cells [45] and activated B cells [46,47]. Treatment of NZB/W models with a combination of metformin and 2DG normalized T cell metabolism and reversed disease biomarkers [45]. The 2DG effectively blocks glucose metabolism of renal macrophages in the diabetic nephropathy model and lupus nephritis model [30,48]. The 2DG treatment also alleviates lipopolysaccharide-induced acute lung injury in a mouse model by suppressing the activation of the NLRP3 inflammasome [49]. Herein, our data showed that 2DG treatment reduces renal infiltrated macrophages in ALD-DNA-driven lupus nephritis. This evidence suggests that macrophages are important targets in mice. Although lactate generation might be dispensable for ALD-DNA-induced production of inflammatory cytokines, 2-DG treatment demonstrated protective efficacy against SLE, indicating that inhibition of glucose metabolism may have positive effects on the treatment of SLE. Meanwhile, systemic treatment with 2DG might target multiple organs, tissues, and cell subsets in vivo, assigning a sophisticated study on the effects of 2DG valuable to advance such translational studies.

In summary, ALD-DNA is able to induce the adaptation of glucose metabolism, profoundly affecting macrophage function (Figure 6). While the glucose metabolism in ALD-DNA-stimulated macrophages is multifaceted, unbalanced glycogenolysis might cut the fuel flux for the PPP, resulting in a ROS-mediated inflammatory response. Accordingly, targeting glucose metabolic adaptation might be a promising strategy for therapeutic explorations in SLE management.

## Data Availability

All data supporting the findings were included in the current study.
